# Squamous Cell Carcinoma of the Gallbladder Presenting as Recurrent Hepatic Abscess: A Case Report

**DOI:** 10.7759/cureus.101639

**Published:** 2026-01-15

**Authors:** Andrés Uribe Valencia, Juan J Zorrilla Ariza, Winny J Barandica Cuaicuan, Lisbeth P Ramirez Perez

**Affiliations:** 1 Surgical Oncology, Universidad del Valle, Cali, COL; 2 Surgery, Universidad CES, Medellin, COL; 3 School of Medicine, Universidad de Caldas, Manizales, COL; 4 School of Medicine, Universidad Icesi, Cali, COL; 5 Pathology, School of Medicine, Universidad del Valle, Cali, COL; 6 Oncology, Universidad Libre, Cali, COL; 7 Internal Medicine, Universidad de Carabobo, Valencia, VEN; 8 School of Medicine, Unidad Central del Valle del Cauca, Tuluá, COL

**Keywords:** bile duct neoplasms, carcinoma, gallbladder neoplasms, immunotherapy, liver abscess, squamous cell

## Abstract

Squamous cell carcinoma (SCC) of the gallbladder and biliary tract is an exceptionally rare and aggressive malignancy that often remains clinically silent until it reaches an advanced stage. We describe the case of a middle-aged man with no significant medical history who initially presented with repeated episodes of hepatic abscesses, each managed as an isolated infectious process. The persistence of symptoms and progressive cholestasis eventually prompted further evaluation, revealing a large hepatobiliary mass with extensive local invasion that could not be surgically resected. Histologic assessment confirmed pure SCC, and the patient was started on systemic therapy with clinical stability during follow-up. This case illustrates an unusual presentation of a rare tumor, in which recurrent hepatic abscesses of bacterial etiology may be related to tumor development. It highlights the importance of considering occult neoplastic processes in patients with hepatobiliary infections and underscores the challenges associated with the diagnosis and treatment of pure biliary SCC.

## Introduction

Gallbladder cancer constitutes the most common malignant neoplasm of the biliary tract [1]. According to Global Cancer Observatory (GLOBOCAN) data, in 2022, its incidence ranked 22nd worldwide, with a total of 122,491 cases and 89,055 associated deaths. Reports of this pathology are far more frequent in Asian populations, which account for over 70% of its incidence, prevalence, and mortality [2]. Most cases correspond to adenocarcinomas, and 1-12% represent squamous variants. However, the prevalence decreases to approximately 0.3-3% when adenosquamous histologies are excluded [3], making “pure” squamous cell carcinoma (SCC) of the gallbladder and biliary tract a rare entity, with much of the available literature limited to case reports. 

This disease is generally asymptomatic and is often identified incidentally during abdominal evaluations; when symptoms are present, they tend to be nonspecific or associated with advanced disease, including ascites, hepatomegaly, a palpable mass in the right abdominal quadrants, obstructive jaundice, vomiting, nausea, food intolerance, and weight loss [3-5]. No clearly defined cause has been established for the development of this type of lesion; however, regardless of its origin, studies consistently indicate that chronic inflammation is the main factor associated with the development of this cancer. Even more infrequently, the disease may present with the formation of a hepatic abscess associated with the tumor mass [3].

We present the case of a male patient with multiple episodes of hepatic abscess who was ultimately diagnosed with locally advanced squamous cell carcinoma of the gallbladder and biliary tract. Furthermore, it is important to highlight that this represents, to the best of our knowledge, the fourth reported case in the scientific literature of a hepatic abscess associated with a tumor mass [3].

## Case presentation

A 41-year-old male patient with no significant past medical history was admitted to a tertiary-care institution after having undergone two percutaneous drainages over the previous five months for hepatic abscesses. Although a bacterial etiology was confirmed in external clinical records, the isolated pathogen was not reported; the patient was managed with both antibiotic and antiparasitic regimens (the first involving segments IVB-V and the second involving segments I, II, and III). It is important to emphasize that the locations of the initial drainages corresponded to the liver segments affected by the tumor lesion. He presented with a four-month history of right upper quadrant pain, nausea, vomiting, generalized jaundice, acholia, and a 20-kg weight loss over the past three months.

On physical examination, the patient was hemodynamically stable, with painful hepatomegaly extending 5 cm below the costal margin and a positive Murphy’s sign. Laboratory tests demonstrated an obstructive cholestatic pattern with elevated total bilirubin at the expense of direct bilirubin, increased alkaline phosphatase and transaminases, mild anemia, no leukocytosis, Tests for hepatitis B, hepatitis C, and human immunodeficiency virus were negative, and blood glucose levels were within the normal range (Table 1).

**Table 1:** Laboratory Results

Test	Patient Value	Reference Range
Total Bilirubin	9.67	0.1 – 1.2 mg/dL
Direct Bilirubin	7.76	0 – 0.2 mg/dL
Hemoglobin	11.6	13.7 – 17.5 g/dL
Leukocytes	6.61	4.23 – 9.07 ×10ˆ³/uL
Alkaline Phosphatase	501	40 – 129 U/L
Alanine Aminotransferase	147	10 – 50 U/L
Aspartate Aminotransferase	76	10 – 50 U/L
Hepatitis B Surface Antigen	0.338	< 0.089 COI
Hepatitis C Antibody	0.03	>1 COI
Human Immunodeficiency Virus 1 and 2 Antibodies	Negative	
Glucose	101	98-107 mmol/L
Glycosylated Hemoglobin	4.6 %	> 5.7 %

**Figure 1:**
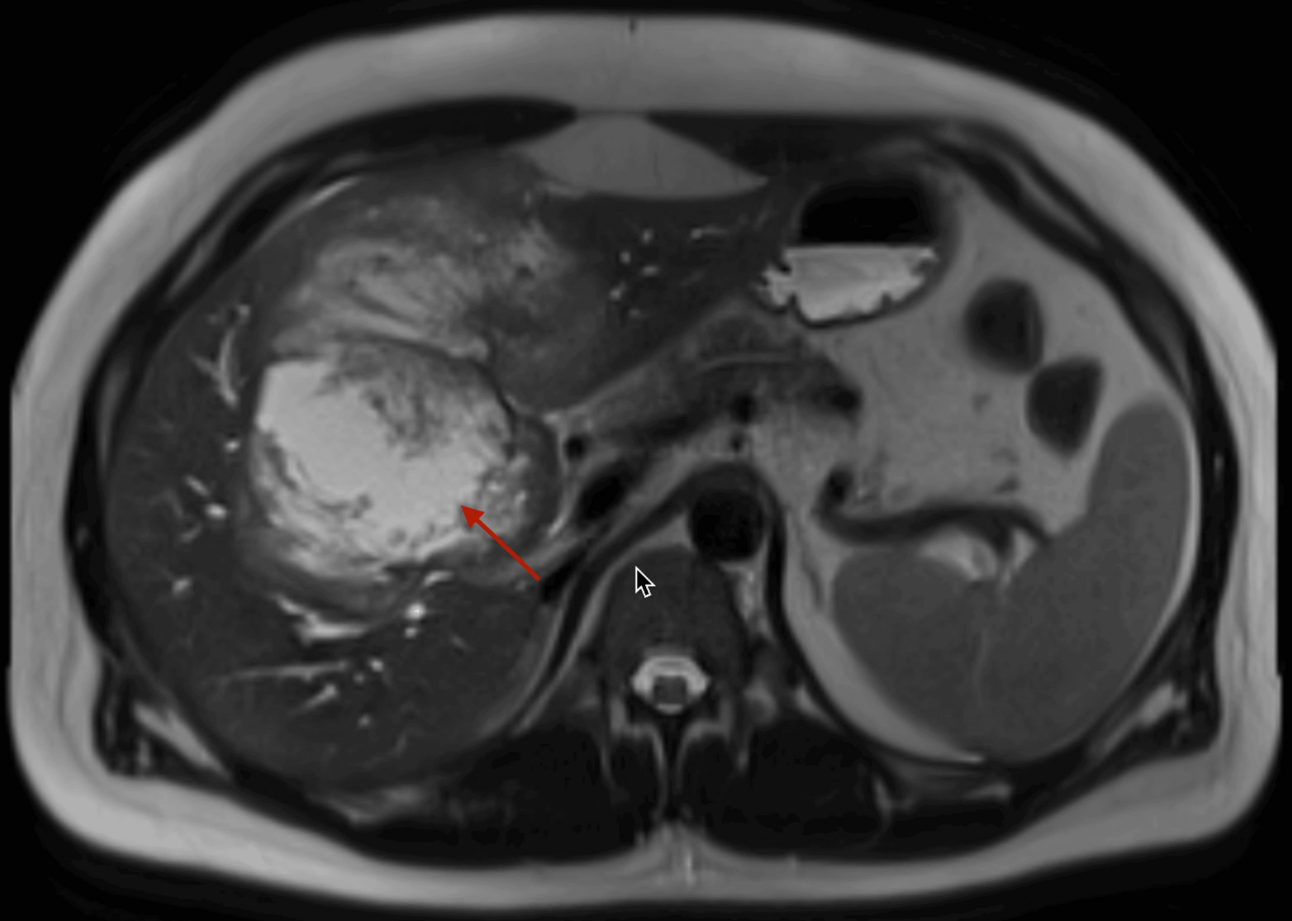
Magnetic resonance cholangiopancreatography The large heterogeneous mass involving hepatic parenchyma from segments IV to VI is indicated by an arrow.

**Figure 2:**
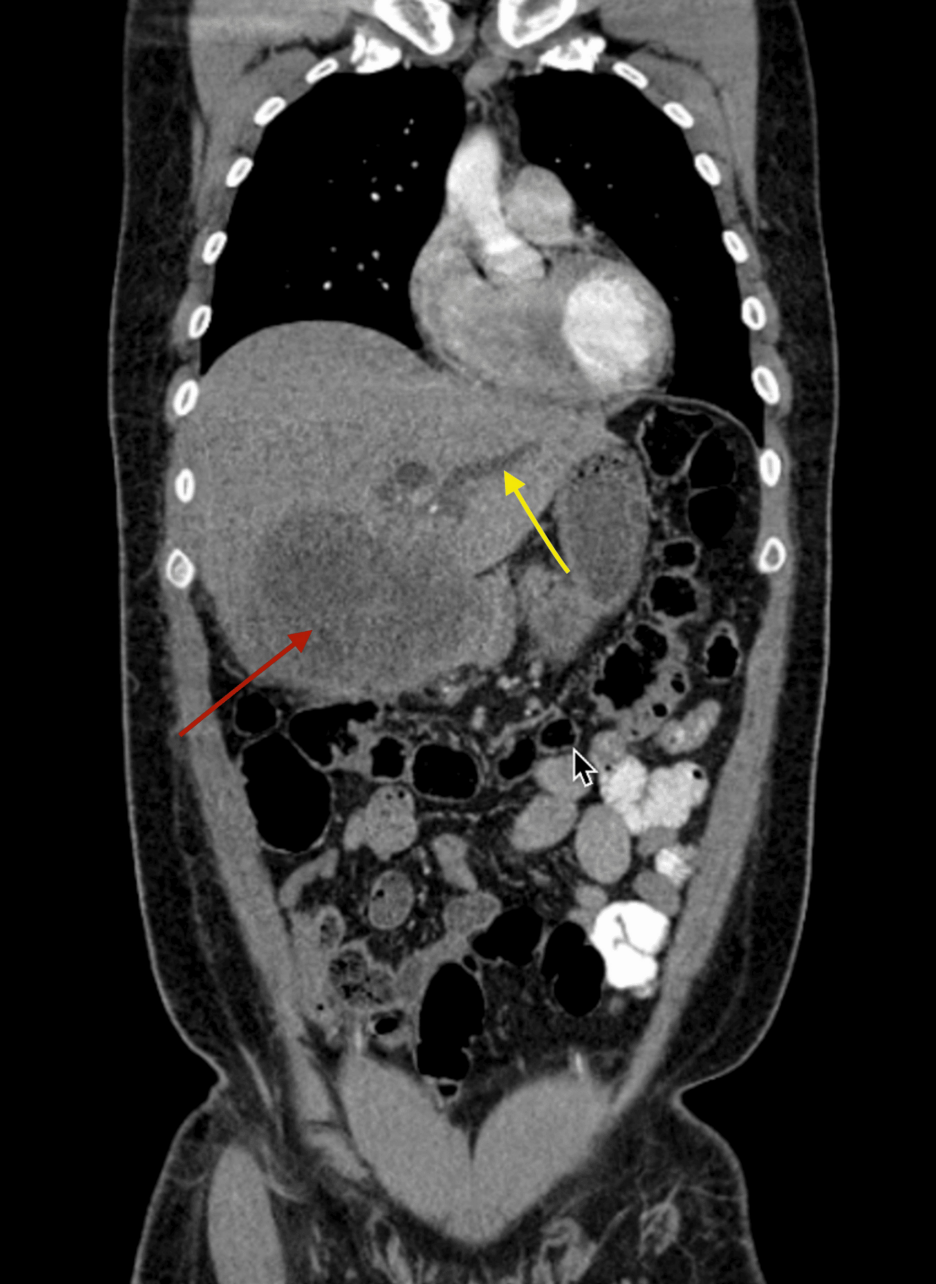
Contrast-enhanced computed tomography of the chest and abdomen In the coronal sections, the intrahepatic mass with ill-defined borders (red arrow), causing marked dilation of the intrahepatic bile ducts (yellow arrow)

An abdominal ultrasound revealed an enlarged liver with heterogeneous echogenicity due to a focal lesion in segment VI measuring 117 × 90 mm with fluid components and irregular borders. A contrast-enhanced abdominal CT scan showed an expansive neoformative lesion measuring 130 × 120 mm involving segments II, IV, and V, associated with severe intrahepatic biliary ductal dilation and no apparent distant secondary involvement. These findings were confirmed by magnetic resonance cholangiopancreatography and contrast-enhanced computed tomography of the chest and abdomen (Figures 1, 2). The patient underwent percutaneous drainage, which revealed bilious, nonpurulent fluid. Culture of the drainage fluid was negative; direct examination for fungi showed no evidence of fungal structures; molecular testing for *Mycobacterium tuberculosis* was negative; and Gram staining demonstrated no leukocytes or bacteria. Subsequently, an endoscopic biliary stent was placed (and later removed).

**Figure 3:**
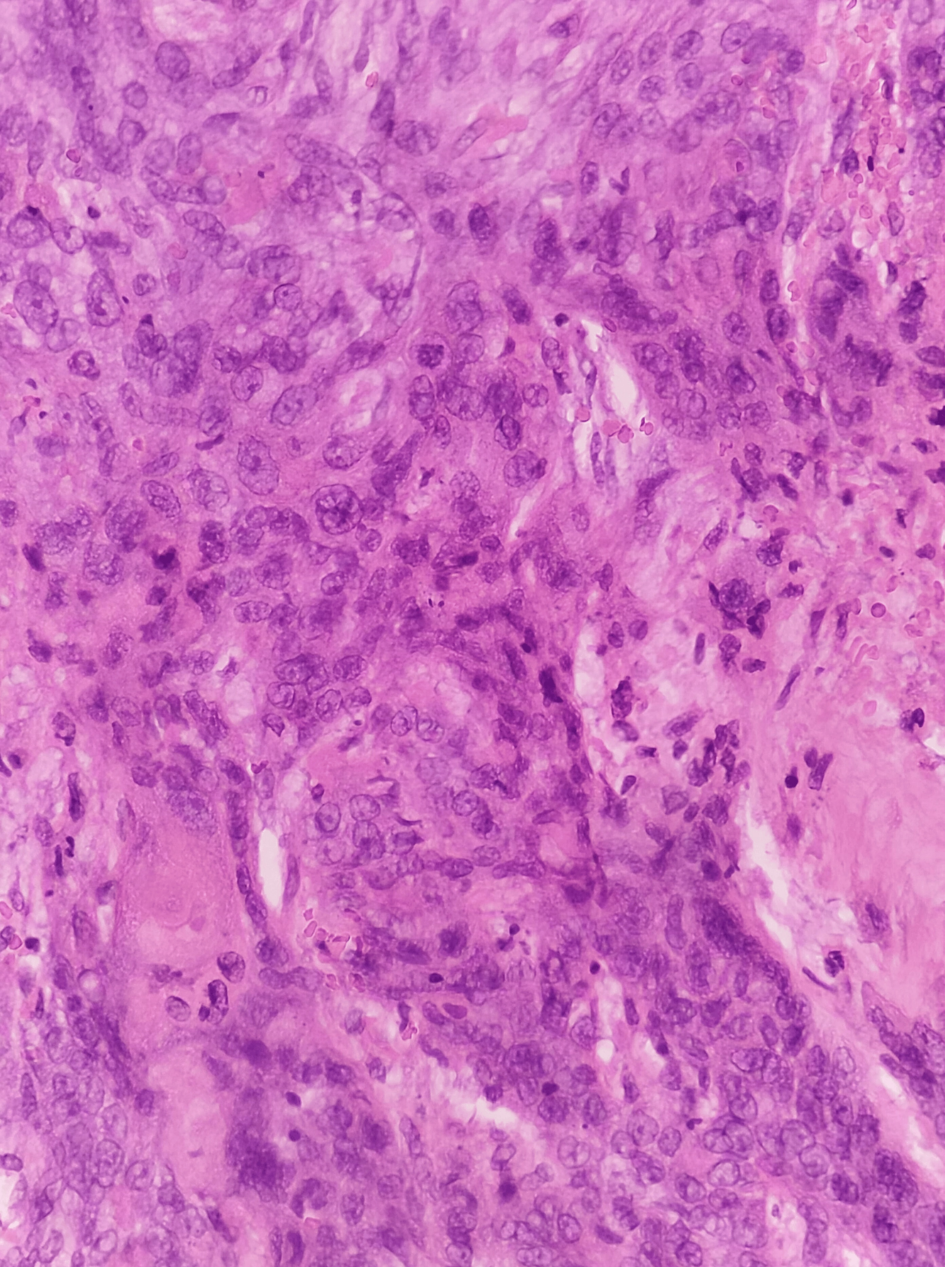
Hematoxylin and Eosin stain number 1 The tumor cells have a polygonal appearance with marked pleomorphism, some displaying conspicuous nucleoli and evident mitotic activity.

**Figure 4:**
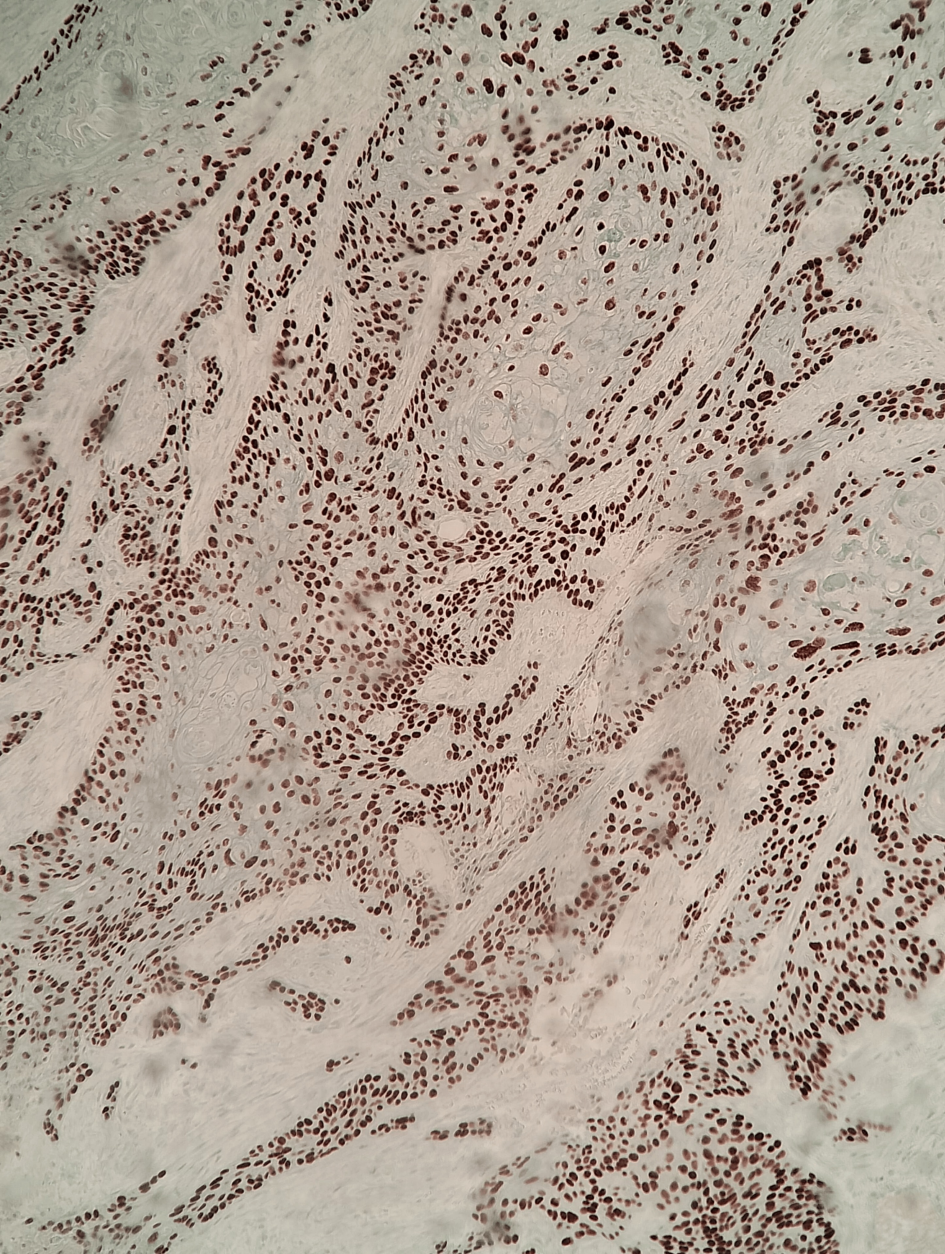
Immunohistochemistry for p40 and p63 Diffuse nuclear positivity for p40 and p63, confirming the diagnosis of squamous cell carcinoma.

**Figure 5:**
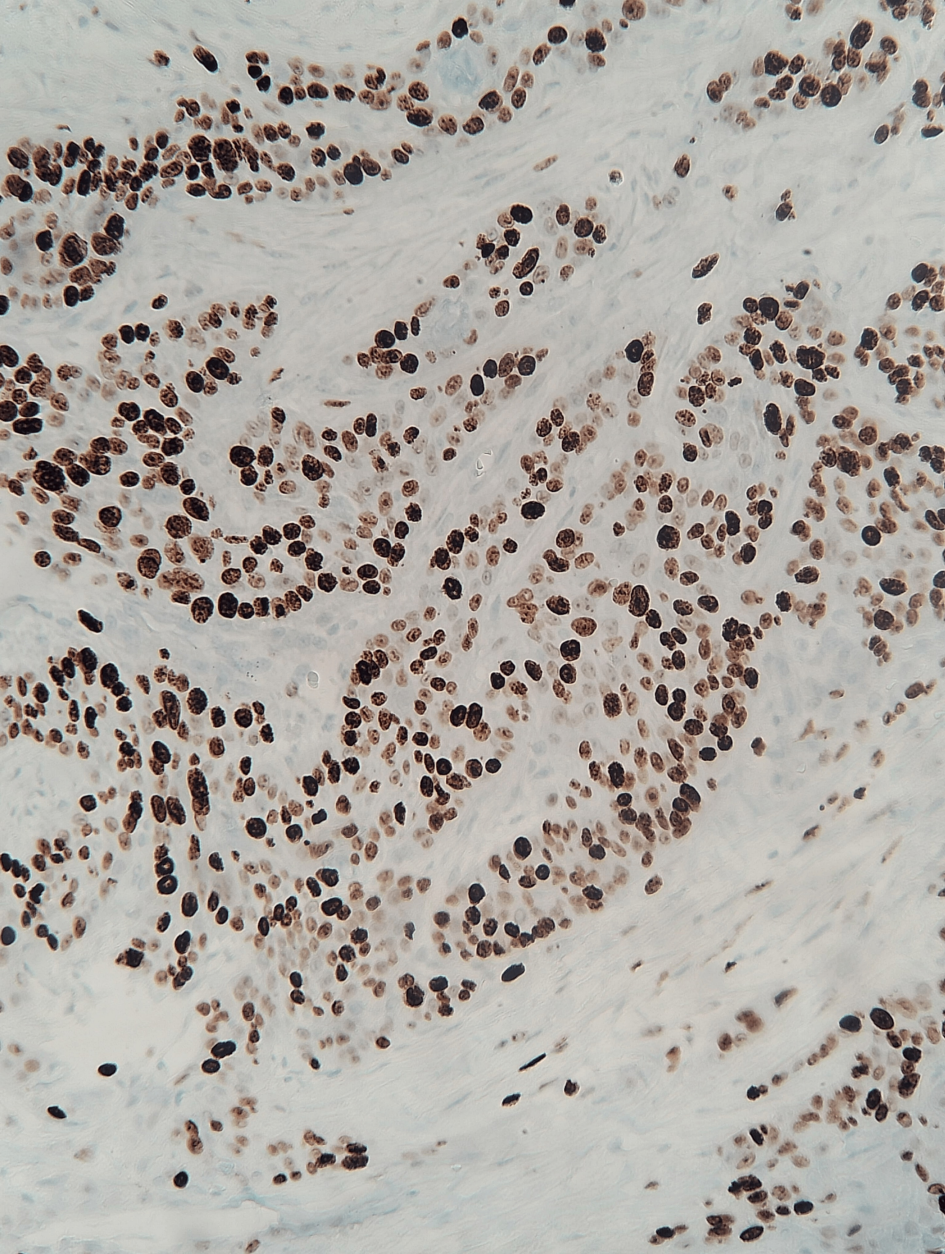
Immunohistochemistry for Ki-67 Ki-67 proliferation index of approximately 60–70%.

**Figure 6:**
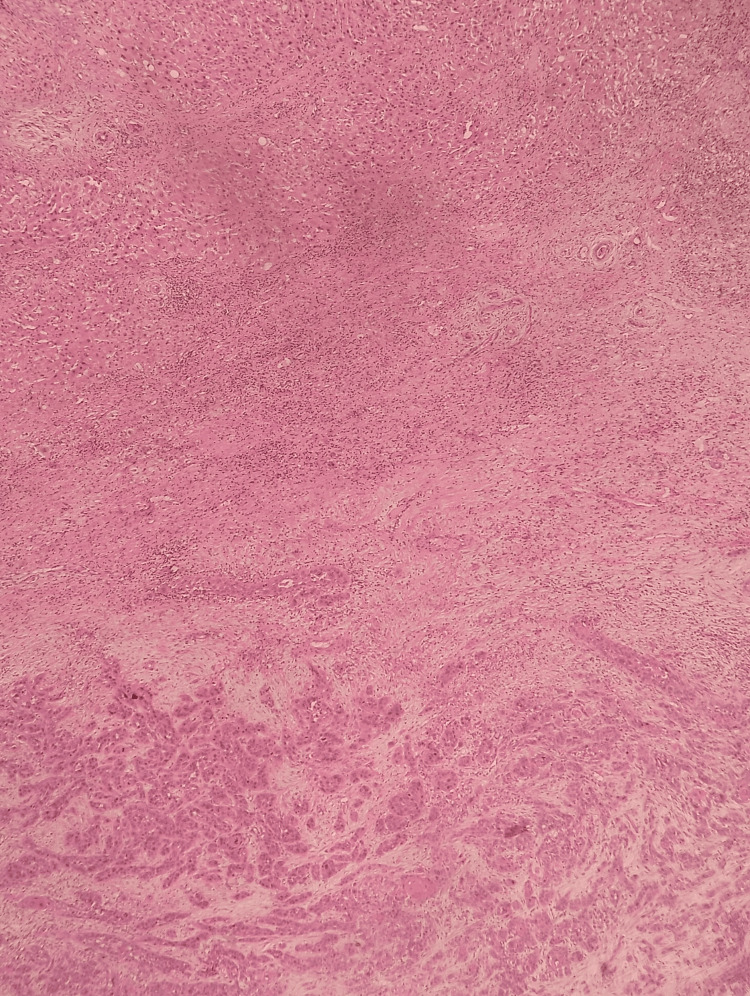
Hematoxylin and Eosin stain number 2 In the upper portion, hepatic parenchyma is observed with hepatocytes showing a reactive appearance, surrounded by a mixed inflammatory infiltrate predominantly mononuclear, and in the lower portion, the transition toward squamous cell carcinoma is seen.

**Figure 7:**
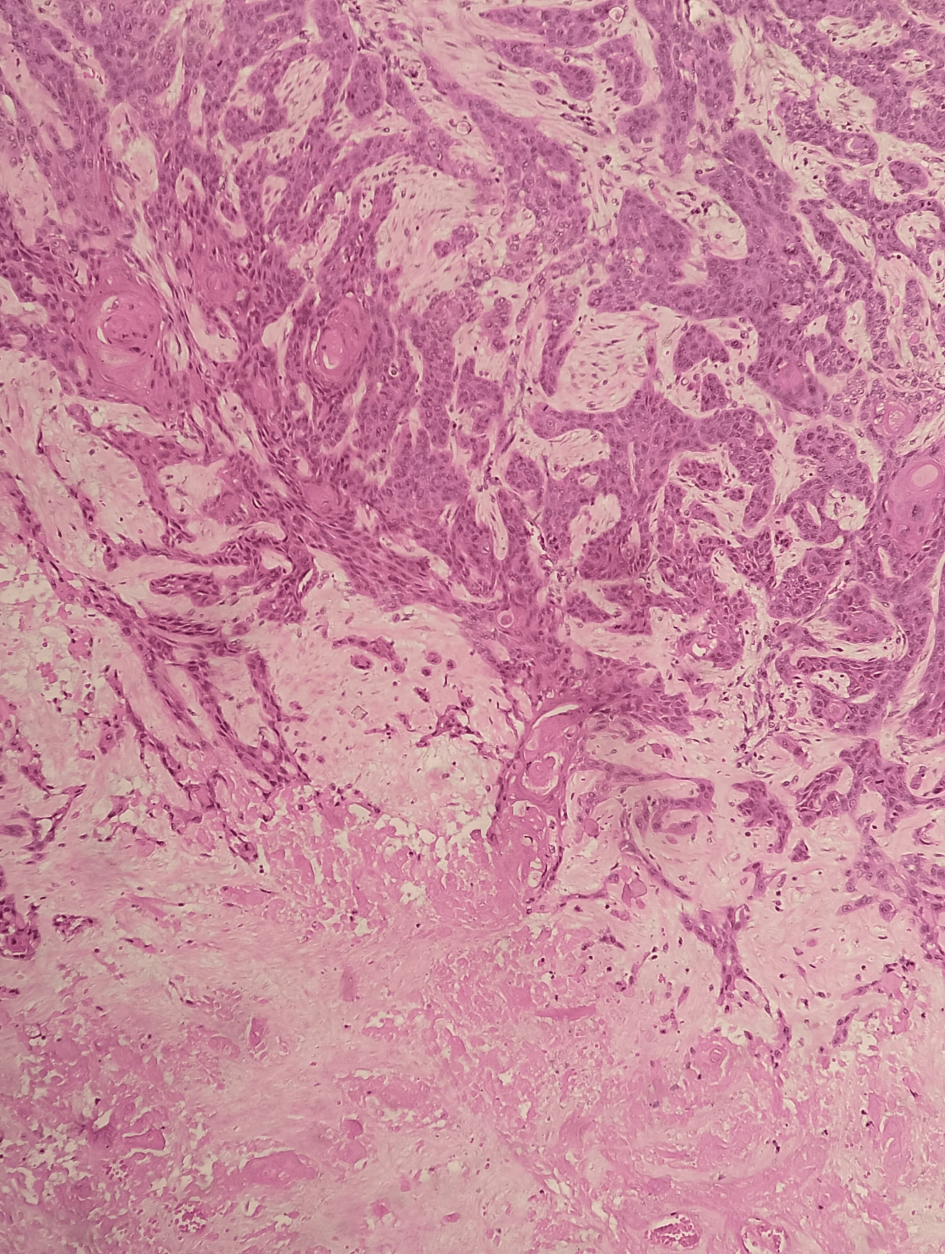
Hematoxylin and Eosin stain number 3 The tumor cells are arranged in nests and trabeculae, infiltrating in a tongue-like pattern and causing desmoplasia of the surrounding stroma, with the formation of keratin pearls

Due to poor clinical improvement and after evaluation by a multidisciplinary team, surgical exploration was recommended. Intraoperatively, a tumor-like mass arising from the gallbladder was identified, with infiltration into the stomach, duodenum, and hepatic hilum, without a clear dissection plane between vascular structures and no viable tissue for reconstruction, rendering complete resection unfeasible. Tissue samples were obtained for histopathological and immunohistochemical analysis (Figures 3-7), which confirmed a squamous cell carcinoma of the biliary tract. The patient was subsequently referred to clinical oncology.

**Figure 8:**
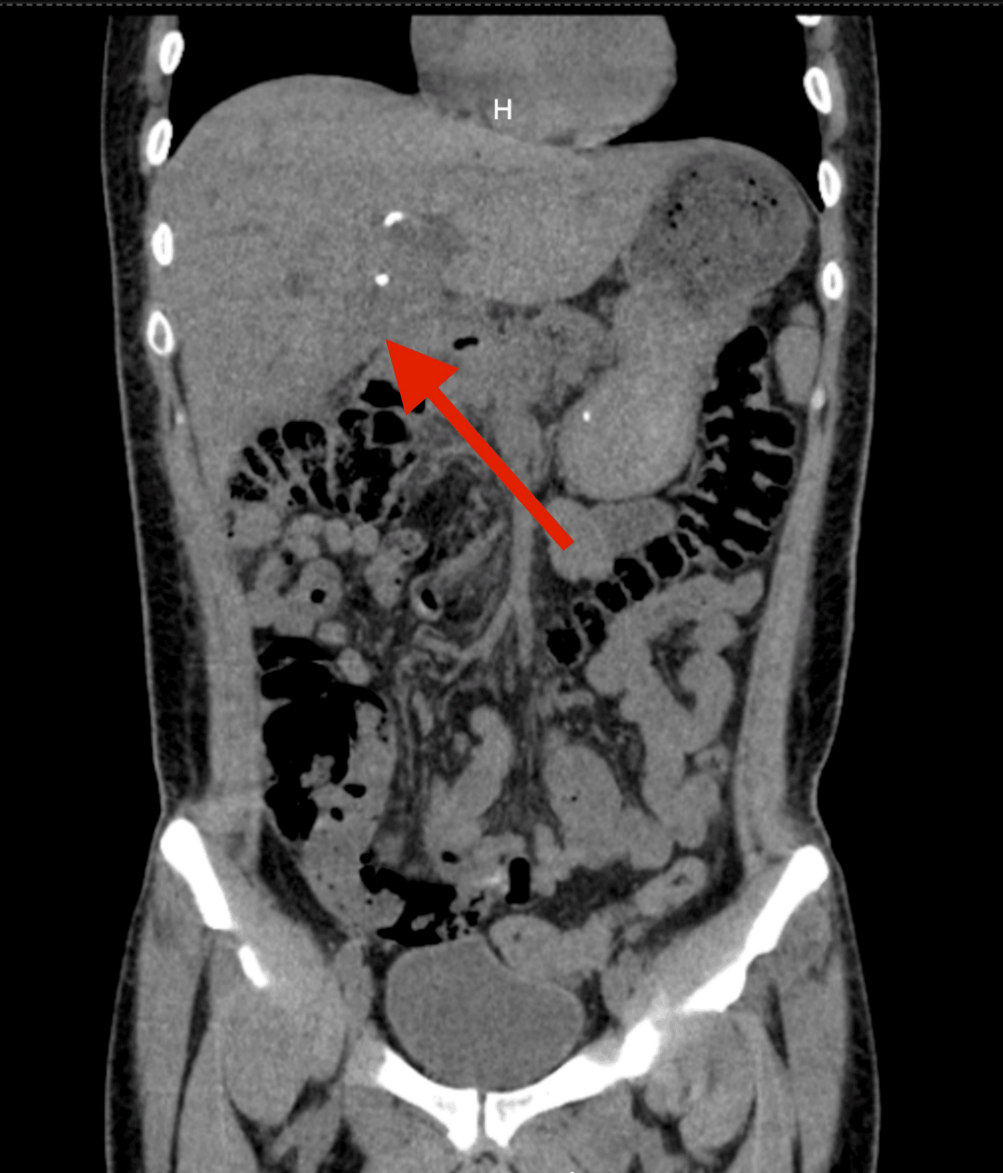
Abdominal and pelvic non-contrast computed tomography The comparative coronal images with the initial scan demonstrate an objective reduction of the tumor lesion, indicated by the red arrow.

Treatment was initiated for a period of seven months with durvalumab, gemcitabine, and cisplatin, which the patient continues to receive to date, showing clinical, biochemical (Table 2), and radiological improvement (Figure 8), as well as good tolerance to the administered therapy.

**Table 2:** Laboratory results follow-up after chemotherapy treatment.

Test	Patient Value	Reference Range
Total Bilirubin	0.60	0.1 – 1.2 mg/dL
Direct Bilirubin	0.40	0 – 0.2 mg/dL
Hemoglobin	8.5	13.7 – 17.5 g/dL
Leukocytes	5.69	4.23 – 9.07 ×10ˆ³/uL
Alkaline Phosphatase	233	40 – 129 U/L
Alanine Aminotransferase	11	10 – 50 U/L
Aspartate Aminotransferase	18	10 – 50 U/L

## Discussion

SCC of the biliary tract is a rare entity, accounting for less than 1% of all reported gallbladder cancers worldwide. Similarly, SCC of the biliary tract (excluding cases with a confirmed gallbladder epicenter) has a comparable incidence, which complicates initial management and treatment [6]. Its clinical presentation is similar to other histologies, with constitutional symptoms, biliary obstruction due to mass effect, and obstructive jaundice being the most commonly reported manifestations in the literature. Like its counterpart, biliary adenocarcinoma, SCC has a higher prevalence among women (female-to-male ratio 3:1) but presents earlier, typically between the fourth and sixth decades of life (the patient in this report falls within this age range) [3,7]. These lesions generally carry a poor prognosis, largely due to late diagnosis when the tumor is already locally advanced.

There is no clear hypothesis regarding the development of this type of lesion. Al-Ramthan et al. [7] describe three possible explanations for its formation, considering that the gallbladder lacks native squamous epithelium [3]. The first theory is malignant transformation of heterotopic squamous epithelium; the second is malignant transformation of metaplastic squamous epithelium; and the third is the development of squamous metaplasia within an adenocarcinoma. Regardless of its origin, authors agree that the main factor associated with the development of this cancer is chronic inflammation [3-10], usually in the form of chronic cholecystitis associated with cholelithiasis. Other potential causes of inflammation include chronic parasitic infections [6], although a clear causal relationship has not been established. This process has been termed the “cancerization of squamous metaplasia of the biliary tract” [10]. Additional risk factors have been described, though with limited evidence, including type 2 diabetes mellitus and hepatitis C virus infection [11].

One of the unusual presentations of this pathology is hepatic abscess formation [3,10]. Due to rapid tumor growth and its typical invasion by contiguity into the hepatic parenchyma, the mass may develop central necrosis, leading to hepatic abscesses that can complicate the diagnostic process. Reviewing the literature, to date, this is the only reported case of a patient experiencing multiple hepatic abscess episodes (three in total), making this an exceptional presentation for an already rare disease.

Surgical management in the early stages is the ideal approach, provided wide resection with adequate margins can be achieved. However, diagnosis is usually late, and overall survival is limited [1,2,11-16]. Roa et al. reported an average survival of 23 months (combined SCC and adenosquamous cases), compared with 50 months for adenocarcinoma [15]. Adjuvant options, as in this patient, are limited. Historically, antimetabolite-based regimens, such as gemcitabine, have been most commonly used [17]. In this particular case, a regimen including durvalumab, gemcitabine, and cisplatin was implemented, based on the TOPAZ-1 trial results, which support the addition of immunotherapy (durvalumab) to standard chemotherapy regimens for advanced biliary tract cancers, showing a 24-month overall survival of 24.9% versus 10.4% in patients without durvalumab. These results were also favorable when comparing objective tumor response, progression-free survival, and the absence of grade 3-4 adverse events between groups [16].

At the time of this report, the patient remains under clinical follow-up, receiving the described therapeutic regimen without associated complications. After four months of treatment, he has shown an objective clinical response as measured by follow-up imaging.

## Conclusions

SCC of both the gallbladder and biliary tract is an uncommon malignancy that requires a well-structured multidisciplinary approach to achieve early and accurate diagnosis, allowing the selection of the most appropriate curative-intent treatment. In patients with advanced-stage disease, as presented in this report, a therapeutic option combining immunotherapy (durvalumab) with chemotherapy (gemcitabine and cisplatin) is supported by current scientific evidence and has demonstrated objective outcomes. We believe that continued investigation into this rare entity will significantly benefit patients, and that case reports with proposed therapeutic strategies contribute to expanding current knowledge and developing improved management approaches.
